# Insights into the LiI Redox Mediation in Aprotic Li–O_2_ Batteries: Solvation Effects and Singlet Oxygen Evolution

**DOI:** 10.1021/acsami.3c12330

**Published:** 2023-12-13

**Authors:** Angelica Petrongari, Vanessa Piacentini, Adriano Pierini, Paola Fattibene, Cinzia De Angelis, Enrico Bodo, Sergio Brutti

**Affiliations:** † Department of Chemistry, Sapienza University of Rome, P.le Aldo Moro 5, Rome 00185, Italy; ‡ Core Facilities, Istituto Superiore di Sanità, Viale Regina Elena 299, Rome 00161, Italy; § CNR-ISC, Consiglio Nazionale Delle Ricerche, Istituto Dei Sistemi Complessi, Rome 00185, Italy; ∥ GISEL - Centro di Riferimento Nazionale per i Sistemi di Accumulo Elettrochimico di Energia, Florence 50121, Italy

**Keywords:** degradation, electrolytes, electron microscopy, electron paramagnetic resonance spectroscopy, Fourier transform infrared spectroscopy, lithium, lithium−oxygen batteries

## Abstract

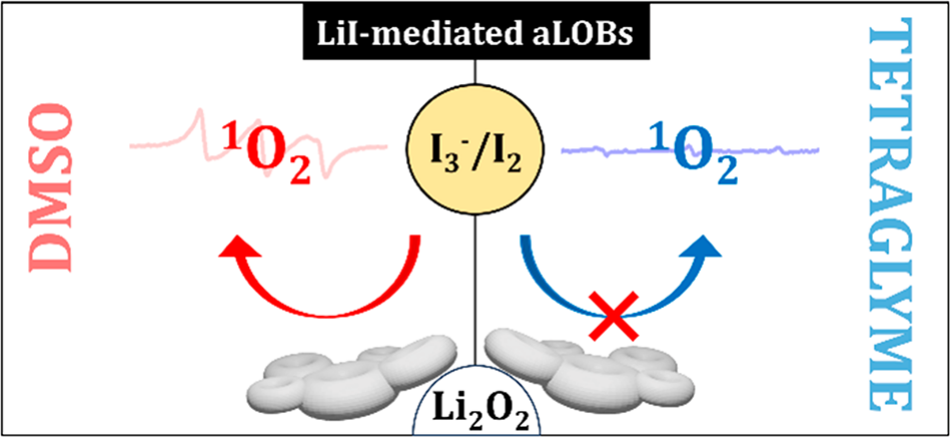

Lithium–oxygen aprotic batteries (aLOBs) are highly promising next-generation secondary batteries due to their high theoretical energy density. However, the practical implementation of these batteries is hindered by parasitic reactions that negatively impact their reversibility and cycle life. One of the challenges lies in the oxidation of Li_2_O_2_, which requires large overpotentials if not catalyzed. To address this issue, redox mediators (RMs) have been proposed to reduce the oxygen evolution reaction (OER) overpotentials. In this study, we focus on a lithium iodide RM and investigate its role on the degradation chemistry and the release of singlet oxygen in aLOBs, in different solvent environments. Specifically, we compare the impact of a polar solvent, dimethyl sulfoxide (DMSO), and a low polarity solvent, tetraglyme (G4). We demonstrate a strong interplay between solvation, degradation, and redox mediation in OER by LiI in aLOBs. The results show that LiI in DMSO-based electrolytes leads to extensive degradation and to ^1^O_2_ release, affecting the cell performance, while in G4-based electrolytes, the release of ^1^O_2_ appears to be suppressed, resulting in better cyclability.

## Introduction

Lithium–oxygen aprotic batteries (aLOBs) are among the most promising systems for next-generation secondary batteries^1^ due to their large theoretical performance (i.e., 3458 Wh/kg).^2^ These energy density figures are due to the use of lithium metal anodes coupled with carbonaceous cathodes where the oxygen reduction reaction (ORR) and the oxygen evolution reaction (OER) occur mediated by the reversible formation of Li_2_O_2_.^3^ Nevertheless, parasitic reactions strongly affect the reversibility and cycle life of Li–O_2_ cells leading to poor rechargeability, electrolyte depletion, and lithium loss.^4−6^ Early studies on the degradation chemistry in aLOBs focused on the nucleophilic character of superoxide and peroxide anions formed during ORR and outlined possible parasitic mechanisms.^7^ However, in recent years, the detrimental key role of singlet oxygen (^1^O_2_) has been clearly demonstrated.^8−10^^1^O_2_ molecules originate from superoxide disproportion during either ORR or OER, during cell discharge and charge, respectively.^11^ Despite the relatively large energy needed to access the ^1^O_2_ channel (∼1 eV above ground state ^3^O_2_), the fact that Li_2_O_2_ oxidation, due to its insulating character and kinetic constraints,^12^ requires overpotentials of more than 1 V enables the ^1^O_2_ release in competition with the conventional ^3^O_2_ evolution, thus leading to premature cell failure.

In order to facilitate the OER kinetics and to reduce overpotentials, many soluble organic^13,14^ or inorganic^15,16^ redox mediators (RMs) have been proposed in the literature. The role of RMs is to catalyze lithium peroxide oxidation by facilitating electron transport between the cathode surface and the Li_2_O_2_ deposits. The RM is first oxidized at the cathode surface via the reaction RM → RM^+^ + e^–^, and the RM^+^ intermediate subsequently oxidizes Li_2_O_2_ as follows: 2RM^+^ + Li_2_O_2_ → 2RM + 2Li^+^ + O_2_.^17,18^ Among the inorganic RMs, lithium iodide is receiving relevant research attention, since it allows very low charge overpotentials and favorable electrochemical performance.^19^

The redox mediation mechanism of LiI in Li–O_2_ cells has been investigated by Qiao et al.^20^ in nonaqueous electrolytes. The LiI redox mediation is delivered by two redox couples, i.e., I^–^/I_3_^–^ and I_3_^–^/I_2_, at increasing potentials above 3.0 V vs Li^+^/Li^0^. Apparently, the presence of traces of water together with LiI promotes the formation of LiOH as the main discharge product, which cannot be oxidized neither by the I^–^/I_3_^–^ nor by the I_3_^–^/I_2_ redox couple upon charge. Thus, the addition of LiI also alters the possible degradation paths promoting the cleavage of the H–OH bond to form LiOH. This reaction path unavoidably enables the H^+^-mediated ^1^O_2_ release.^19,21−23^

The use of LiI in aprotic LOBs has been studied focusing on performance analysis: as a consequence, the understanding of the redox mediation of LiI is controversial. In particular, the ability of the I_3_^–^ species to oxidize lithium peroxide is still under debate.^20,22,24^ Solvent effects in the oxidizing power of I_3_^–^ or I_2_ species have been investigated by Nakanishi et al.^25^ and Leverick et al.;^26^ both groups observed enhanced OERs in polar solvents like dimethyl sulfoxide (DMSO). Apparently, solvents with large dielectric constants boost the OER thermodynamic driving force thanks to the increase of the redox potential of the I_3_^–^/I^–^ couple. This thermodynamic push is likely due to stronger solvation of the I^–^ anion.

The introduction of LiI in the Li–O_2_ cell formulation rises additional concerns for its impact on degradation processes: it has been recently reported by Wang et al.^27^ that the formation of IO_3_^–^ anion during cell operation could be a major source of solvent deprotonation in a large variety of electrolytes. However, as far as we know, an experimental comparative analysis of the impact of the solvent polarity on the degradation chemistry and on ^1^O_2_ release in LiI-mediated aprotic LOBs has not been attempted so far.^8^

Here, we demonstrate the strong interplay between solvation, degradation, and the redox mediation in the OER by LiI in aLOBs. We tested LiI as RM additive in dimethyl sulfoxide (DMSO)- and tetraglyme (G4)-based electrolytes and verified the performance of aLOBs in static conditions with no excess of O_2_. Extensive degradations are observed in DMSO, whereas G4 electrolytes deliver reversible cycling. The different performance is driven by the selective ^1^O_2_ release in DMSO, confirmed experimentally by electron paramagnetic resonance (EPR) using 4-oxo-TEMP as ^1^O_2_ trap.^28,29^ On the opposite side, ^1^O_2_ is apparently suppressed in the G4-based electrolyte, thus demonstrating the key role played by solvation on the thermodynamics of parasitic chemistry in redox mediated LOBs.

## Experimental Methods

### Electrolyte Preparation

High-purity tetraglyme [tetraethylene glycol dimethyl ether, anhydrous, ≥99%] and DMSO [dimethyl sulfoxide, anhydrous, ≥99%] were purchased from Sigma-Aldrich and dried with 3 Å molecular sieves for at least 1 week before use. Battery grade LiTFSI (lithium bis(trifluoromethanesulfonyl)imide extra dry <20 ppm of H_2_O, Solvionic) and LiI (lithium iodide, AnhydroBeads, 99%, Sigma-Aldrich) were used as received. The two electrolyte formulations consist of 1 M LiTFSI + 200 mM LiI in tetraglyme and DMSO.

### Electrochemical Measurements

An EL-CELL ECC-Air test cell designed for Li–O_2_ tests in aprotic electrolytes was used to perform electrochemical experiments. Precut discs of a commercial carbonaceous GDL (MTI Corp.) were used as cathodes. A metallic lithium foil was used as a negative electrode. A nickel foam disc (16 mm diameter) was used above the GDL to ensure a homogeneous O_2_ impregnation. A glass fiber separator (Whatman, 1.55 mm thickness, 18 mm diameter), soaked in 1 M LiTFSI + 200 mM LiI in DMSO or G4 electrolytes, was used. Cell assembly was performed in an Ar filled glovebox (Iteco Eng SGS-30, H_2_O < 0.1 ppm). The Li–O_2_ cells were filled with pure O_2_, setting a static final pressure of 2.0 bar in the cell volume (head space 4.3 cm^3^). Galvanostatic cycling tests were run on (−)Li^0^|LiTFSI 1 M + LiI 200 mM in DMSO|GDL(+) and (−)Li^0^|LiTFSI 1 M + LiI 200 mM in G4|GDL(+) cells at 0.1 mA cm^–2^ with a limited capacity of 0.2 mAh cm^–2^ and cutoff potentials of 2.0 and 3.6 V vs Li^+^/Li^0^, using a galvanostat MTI 8-channel Battery Analyzer. Coulombic Efficiency (CE) is calculated as the ratio between the capacity achieved in discharge and the capacity achieved in charge.

### Physical–Chemical Characterization

Electrodes for postmortem studies were washed twice in fresh DMC and then dried in vacuum. ATR-FT-IR spectra were acquired using a Bruker Lumos II microscope with an integrated FT-IR spectrometer suited for sample analysis in attenuated total reflection using a Ge crystal. A resolution of 4 cm^–1^ and 10 scans were set. Raman spectra were registered using a DILOR LabRam confocal micro-Raman instrument equipped with a He–Ne laser source at 632.7 nm. Samples were held in 3D-printed sample holders sealed with a cover glass window and nail polish in order to avoid undesired reactions with air moisture. An HR-FESEM Zeiss Auriga coupled with a Bruker EDX system was used for ex situ morphological characterization and elemental mapping of electrodes.

### EPR Measurements

The spin trap 4-oxo-TEMP (2,2,6,6-tetramethyl-4-piperidone, 95%) and its oxidized form 4-oxo-TEMPO were purchased from Sigma-Aldrich and used as received. Samples were prepared in an Ar filled glovebox (Iteco Eng SGS-30, H_2_O < 0.1 ppm) at room temperature. Standard solutions were prepared at concentrations of 5, 50, and 100 mM of 4-oxo-TEMPO in DMSO. Reaction solutions of I_2_ 50 mM + LiI 100 mM + 80 mM 4-oxo-TEMP in DMSO and G4 were prepared, and excess Li_2_O_2_ was added shortly before starting the EPR measurements. 50 μL of the as-prepared dispersions were held in Suprasil quartz tubes of 2 mm internal diameter. The tubes were sealed with low-impurity wax to avoid undesired contact with air moisture. EPR spectra were recorded at room temperature with a continuous wave X-band spectrometer (Bruker ELEXSYS) equipped with a high sensitivity microwave cavity (Bruker SHQ). EPR acquisition parameters were: 2 mW microwave power, 0.02 G modulation amplitude.

### Computational Details

The IR spectra of isolated molecules used to assign the spectra were calculated using density functional theory (DFT) at wB97XD/def2-TZVP^30^ with the Orca package distribution, version 5.03.^31^ The molecular geometries were optimized to the energy minimum, and the IR spectra were obtained by a standard harmonic frequency analysis.

The EPR spectra were simulated and best fitted with Easyspin, a Matlab-based software.^32^

## Results

### Electrochemical Measurements

**Figure 1:**
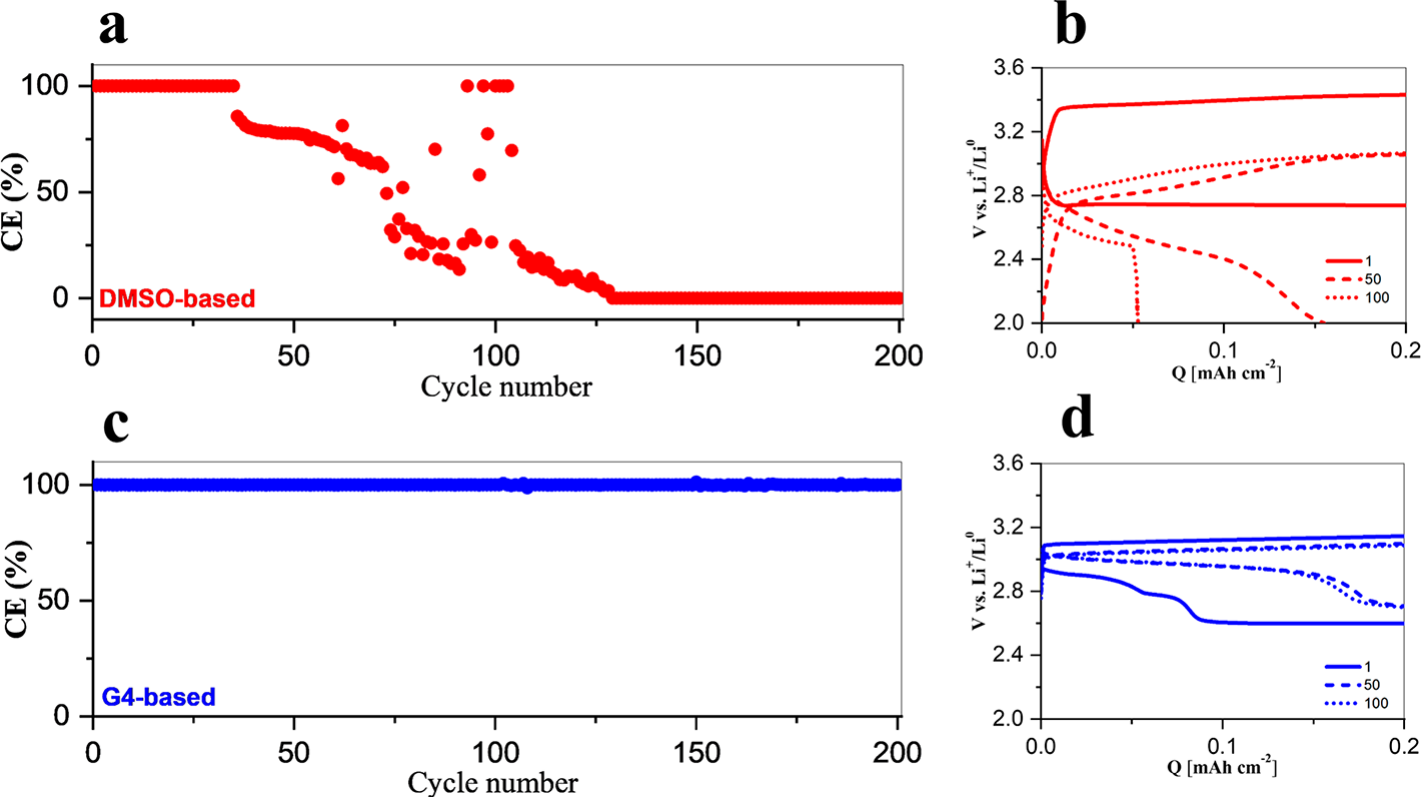
Coulombic efficiencies of Li–O_2_ cells cycling at *J* = 0.1 mA cm^–2^, *Q*_lim_ = 0.2 mA cm^–2^, for 200 cycles with (a) 200 mM LiI + LiTFSI 1 M in the DMSO electrolyte and (c) 200 mM LiI + LiTFSI 1 M in the G4 electrolyte. Voltage discharge and charge profiles at the 1st, 50th, and 100th cycle with (b) DMSO-based electrolyte and (d) G4-based electrolyte.

The performance of aLOBs assembled using DMSO- or G4-based electrolytes are shown in Figure 1 in terms of Coulombic efficiencies upon cycling (Figure 1a,c). Apparently, the cycling performance of LiI-mediated Li–O_2_ cells is strongly modified by the solvent used in the electrolyte.

Cell formulations based on the DMSO electrolyte (Figure 1a) can work reversibly for ∼35 cycles only, followed by a scattered decrease of the Coulombic efficiency. This performance decay is driven by a monotonic decrease of the discharge capacity (see Figure 1b), possibly originating from the lack of molecular oxygen. The potential profiles in Figure 1b, ranging from the first to the 100th cycle, show that independent from discharge capacity the charge curve always reaches the capacity limit. Moreover, the mean charge potentials observed at the 50th and 100th cycle is significantly below that of the first cycle, showing values smaller than the thermodynamical redox potential of the couple Li_2_O_2_/O_2_ (i.e., 2.96 V vs Li^+^/Li)^33^ for more than 50% of the entire charge. This suggests that a change in the electrochemical process during charge occurred upon cycling, deviating from the desired process, i.e., I^–^ oxidation to I_3_^–^, which then promotes Li_2_O_2_ oxidation. The strong modification of the charge process likely involves parasitic reactions driven by singlet oxygen.

Conversely, in the case of the G4-based electrolyte, cells keep good performance for at least 200 cycles (Figure 1c) despite some alterations in the voltage profiles upon cycling (Figure 1d). The charge potential profile always below 3.2 V vs Li is related to the oxidation reaction 3I^–^ → I_3_^–^ + 2e^–^, that occurs in parallel to the simultaneous oxidization of Li_2_O_2_ by I_3_^–^. Overall, a redox mediated OER occurs, pushed by the thermodynamic driving force of the RM.^34^ However, the discharge profiles modify upon cycling: in particular, two-step profiles are observed at the 50th and 100th cycles. This evidence can be due to the accumulation of I_3_^–^ upon cycling: in fact, I_3_^–^ can be reduced to I^–^ during discharge at around 3.0 V, while the second plateau at around 2.8 V is likely related to the ORR. This behavior has been previously observed in the literature,^25,35^ and it is due to the slow oxidation kinetics of Li_2_O_2_ by I_3_^–^ in ethereal solvents.^26^ Therefore, I_3_^–^ accumulates upon charge and can be reduced upon discharge in competition to the ORR. Under this hypothesis, the measured discharge capacity is only partially accountable for the Li_2_O_2_ formation as well as charge capacity cannot be fully due to the Li_2_O_2_ oxidation. A quantitative evaluation of the amount of Li_2_O_2_ electrochemically formed and/or dissolved during discharge and/or charge is still experimentally challenging: additional methodological innovations are necessary to reliably provide quantifications of the amount of lithium peroxide over electrodes. Thus, using our experimental approach and equipment, we cannot decouple the two contributions to the total discharge capacity. Qualitative evaluations are reported in the Supporting Information based on simple stoichiometric considerations.

Turning to the charge step, in the literature, Kwak et al.^16^ and Bi et al.^19^ discussed the possible suppression of OER induced by the most favorable I^–^/I_3_^–^ and I_3_^–^/I_2_ oxidations. However, in our case, simple stoichiometric considerations unavoidably prove the occurrence of the OER upon charge (see Figure S1). In particular, at least 44% of the cumulative capacity exchanged upon charge must originate from the oxidation of Li_2_O_2_ to release ^3^O_2_. Thus, it is necessary to specify that a prolonged cycle life of the Li–O_2_ cell with this electrolyte can be, in part, accountable to iodide chemistry and not only to an optimization of ORR/OER processes. However, many strategies are being proposed in the literature to suppress the contribution of iodine redox couples to battery capacity, and the preliminary results reported suggest that, with a suitable cell configuration, LiI/G4 electrolytes can provide interesting ORR/OER performances.^35,36^ Besides the possible contribution provided by the I_3_^–^/I^–^ couple, which likely contributes in both cell formulations, we argue that these promising results in the case of the LiI/G4 electrolyte originated from the suppression of the singlet oxygen evolution, as we will discuss below.

### Postmortem Characterization of Electrodes

**Figure 2:**
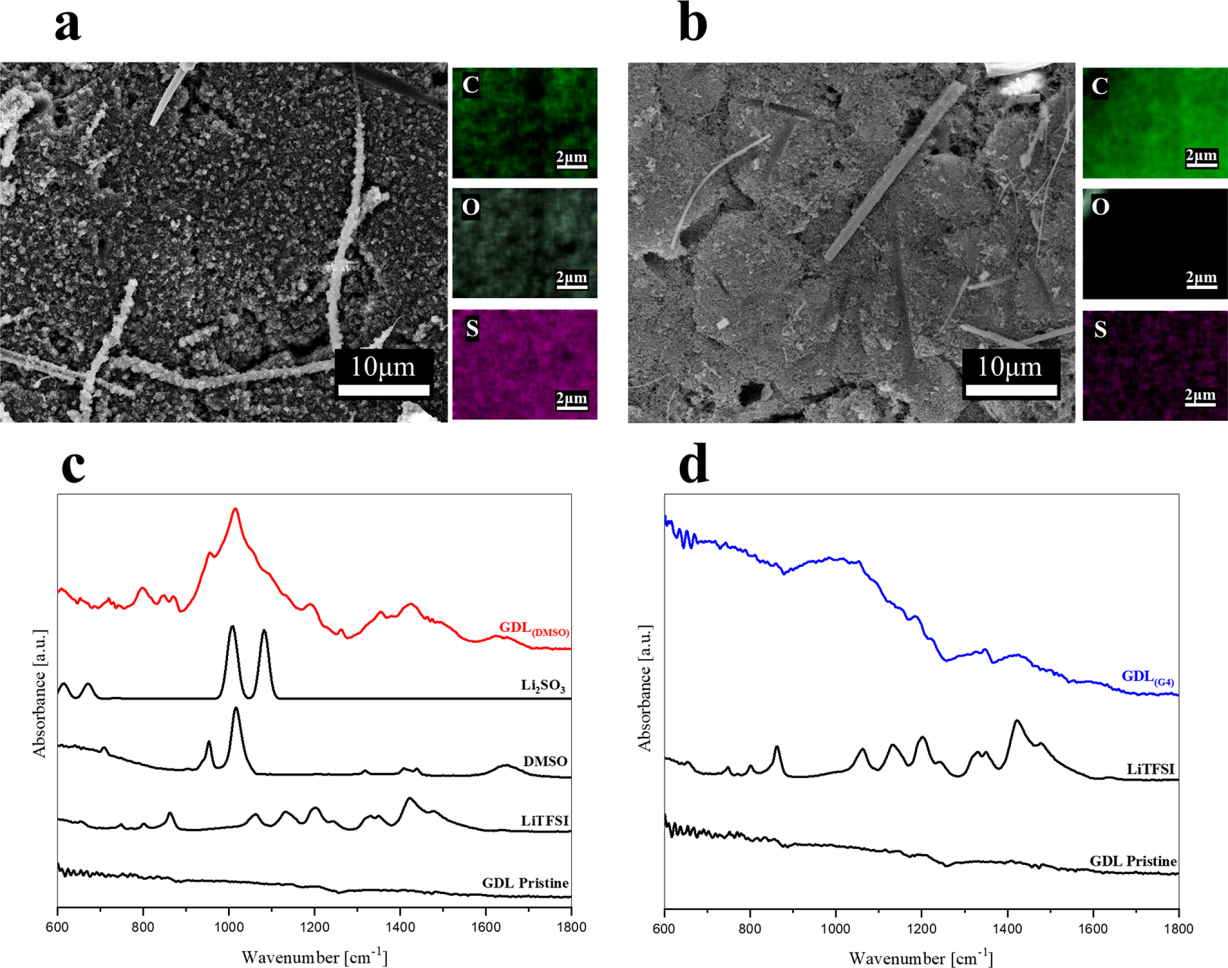
(a) SEM micrograph of postmortem gas diffusion layer cycled with the DMSO-based electrolyte for 200 cycles, GDL_(DMSO)_, at 5000× magnification. (b) SEM micrograph of postmortem gas diffusion layer, cycled with the G4-based electrolyte for 200 cycles, GDL_(G4)_, at 5000× magnification. (c) ATR-FTIR spectrum GDL_(DMSO)_. Reference spectra of Li_2_SO_3_ (theoretical), DMSO, LiTFSI, and pristine GDL. (d) ATR-FTIR spectrum GDL_(G4)_. Reference spectra of LiTFSI and pristine GDL.

The experimental evidence of cell failures in the case of DMSO-based electrolytes registers in the evolution of the surface composition at the positive and negative electrodes during cycling. To confirm this point, we performed postmortem analyses of the GDLs and lithium counter-electrodes collected postmortem from cells after cycling in the two different electrolytes. SEM/EDX and ATR-FTIR data sets on samples collected from the DMSO-based electrolyte (GDL_(DMSO)_) and the G4-based electrolyte (GDL_(G4)_) are shown in Figure 2. EDX quantitative analysis on GDL samples is reported in Table 1.

**Table 1:** EDX Elemental Quantitative Analysis on GDL Cycled with the DMSO-Based Electrolyte for 200 Cycles and GDL Cycled with the G4-Based Electrolyte for 200 Cycles

	atomic %					
sample	C	O	S	F	Ni	I
GDL_(DMSO)_	38.12	24.93	16.10	6.52	9.84	4.49
GDL_(G4)_	94.9	1.3	0.2	2.4	0.8	

SEM micrographs and EDX elemental quantification confirm that the electrolyte strongly impacts both electrode morphology and composition after prolonged cycling in DMSO. The GDL_(DMSO)_ surface (Figure 2a) is covered with distinguished deposits that are likely constituted by electrolyte degradation products. Large amounts of sulfur distributed on the cathode surface result from the EDX analysis reported in Table 1 and in the elemental mapping, hinting that extended degradation of DMSO and LiTFSI occurred. This is confirmed by the ATR-FTIR spectrum reported in Figure 2c, where the main signals are in the range between 1000 and 1100 cm^–1^ that corresponds to the S–O stretching region.^37,38^ The spectrum in this region in part resembles that of DMSO but likely also involves other sulfur compounds coming from its decomposition and, to a minor extent, LiTFSI decomposition. The presence of lithium sulfite cannot be excluded also considering the ex situ Raman spectrum (Figure S2). This evidence is in line with previous literature reports that identified Li_2_SO_3_ as one of the degradation products of DMSO during ORR, due to direct reaction with lithium superoxide.^39^ It is reasonable to hypothesize that a similar degradation path can also be induced by singlet oxygen that is strongly enhanced in this electrolyte. Moreover, since a non-negligible amount of iodine is found on GDL_(DMSO)_, it is likely that also the strong nucleophile IO_3_^–^ plays a role in the degradation of DMSO, in agreement with the recent report of Wang et al.^27^ that demonstrates the possibility of DMSO spontaneous deprotonation by IO_3_^–^. It is worth noticing that, based on Wang et al.’s calculations, IO_3_^–^ formation is strongly triggered by singlet oxygen evolution.

On the contrary, SEM/EDX characterization of the GDL sample (Figure 2b) collected after cycling in the G4-based electrolyte suggests the presence of a thin CEI (cathode–electrolyte interphase) layer. In fact, from the EDX quantification reported in Table 1, it can be observed that the GDL_(G4)_ surface is primarily constituted by carbon. Only very small amounts of sulfur and fluorine were detected on the surface, possibly resulting from moderated LiTFSI degradation. The corresponding ATR-FTIR spectrum (Figure 2d) is constituted mainly by low intensity LiTFSI features and a band in the region of C–O–C stretching,^40^ between 800 and 1100 cm^–1^, that is likely due to G4 degradation. Although signals in the range of 900–1100 cm^–1^ can also be attributed to S–O bonds, their contribution is likely negligible based on the low content of sulfur detected by the EDX quantitative analysis (Table 1). These results are compatible with the formation of a natural CEI layer, and different from the DMSO case, the occurrence of severe degradations of electrolyte can be excluded. Moreover, EDX reports a very low content of oxygen on the electrode surface, suggesting that no accumulation of Li_2_O_2_ due to inefficient oxidation has occurred during cycling.

**Figure 3:**
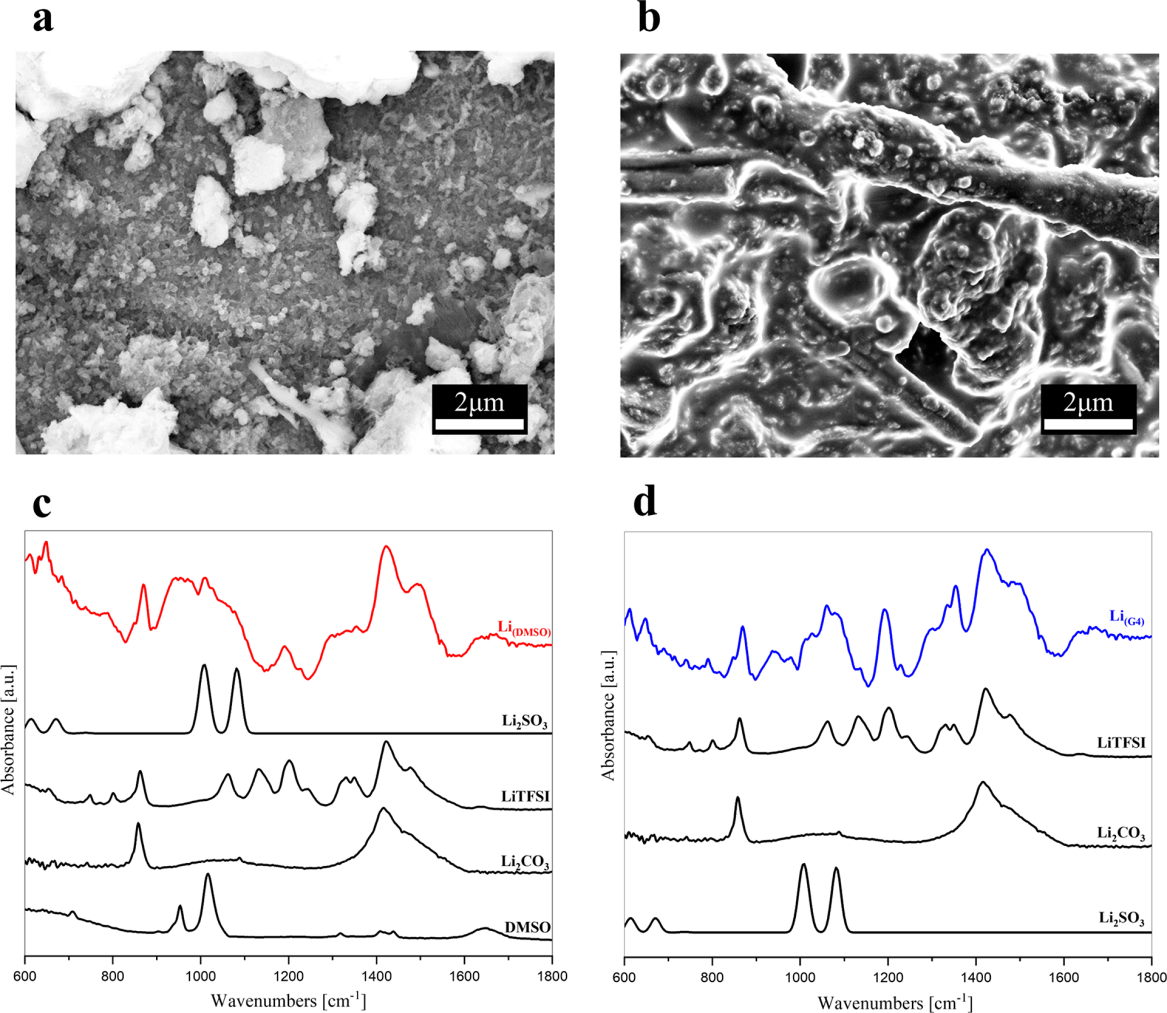
(a) SEM micrograph of postmortem Li metal cycled with the DMSO-based electrolyte for 200 cycles, Li_(DMSO)_, at 20000× magnification. (b) SEM micrograph of postmortem Li metal cycled with the G4-based electrolyte for 200 cycles, Li_(G4)_, at 20000× magnification. (c) ATR-FTIR spectrum of Li_(DMSO)_. Reference spectra of Li_2_SO_3_ (theoretical), LiTFSI, Li_2_CO_3_, and DMSO. (d) ATR-FTIR spectrum of Li_(G4)_. Reference spectra of LiTFSI, Li_2_CO_3_, and Li_2_SO_3_ (theoretical).

The corresponding characterization of lithium metal anodes is shown in Figure 3. Both Li_(DMSO)_ and Li_(G4)_ show an uneven morphology of the lithium surface with large differences. In the case of Li_(DMSO)_, degradation products form a thick SEI layer in a film-like morphology (Figure 3a).

The EDX elemental analysis reported in Table 2 indicates oxygen and carbon as the main components, together with smaller amounts of sulfur. The absence of fluorine from the EDX analysis of this sample suggests that the majority of degradation products containing sulfur likely come from DMSO. The elemental analysis of Li_(DMSO)_, as well as that of GDL_(DMSO)_, also reports a non-negligible percentage of Ni. Ni foam is employed in the cell configuration at the cathode side (see Experimental Methods) to promote oxygen diffusion, and it is expected to be inert under the cell working conditions. The presence of Ni on the anode side thus hints at a remarkable corrosion of the Ni foam during the cell operation, possibly promoted by the ROS (reactive oxygen species).

**Table 2:** EDX Elemental Quantitative Analysis on Li Metal Cycled with the DMSO-Based Electrolyte for 200 Cycles and Li Metal Cycled with the G4-Based Electrolyte for 200 Cycles

	atomic %				
sample	C	O	S	F	Ni
Li_(DMSO)_	22.55	40.46	3.23		6.74
Li_(G4)_	36.48	24.64	9.22	24.07	0.22

The ATR-FTIR spectrum of Li_(DMSO)_ (Figure 3c) shows strong contributions by sulfur compounds in the region of 1000–1100 cm^–1^, similar to the case of GDL_(DMSO)_, likely derived from the DMSO degradation. The spectrum of this sample is also compatible with the presence of Li_2_SO_3_. The LiTFSI–CF_3_ peak^41,42^ at 1190 cm^–1^ can also be clearly identified, indicating that, although the EDX analysis could not detect fluorine, LiTFSI fragments are present on the electrode surface. The remaining main features in the Li_(DMSO)_ ATR-FTIR spectrum are ascribed to lithium carbonate, but its contribution is necessarily overestimated because of the previous exposure of the sample to CO_2_ during the sample loading in the SEM apparatus.

A different picture emerges when observing Li_(G4)_. The SEM micrograph (Figure 3b) shows the typical aspect of lithium metal in a dendrite-like morphology, suggesting the presence of a thin SEI layer. In the ATR-FTIR spectrum (Figure 3d), the most relevant spectral features are related to LiTFSI and Li_2_CO_3_. Additionally, signals in the C–O–C stretching region are detected. It is likely that also a contribution from Li_2_SO_3_ is present, as a result of LiTFSI degradation, in agreement with previous findings.^43^ The EDX elemental analysis reported in Table 2 indicates high quantities of sulfur and fluorine, confirming the predominance of LiTFSI fragments in SEI composition. Overall, the effect of DMSO solvation on LiI redox mediation has a detrimental impact on the stability of cell components, leading to extended side reactivity that affects the cell cyclability.

### Chemical Reactivity of Lithium Peroxide with Iodine: White Light Images and EPR Spectra

**Figure 4:**
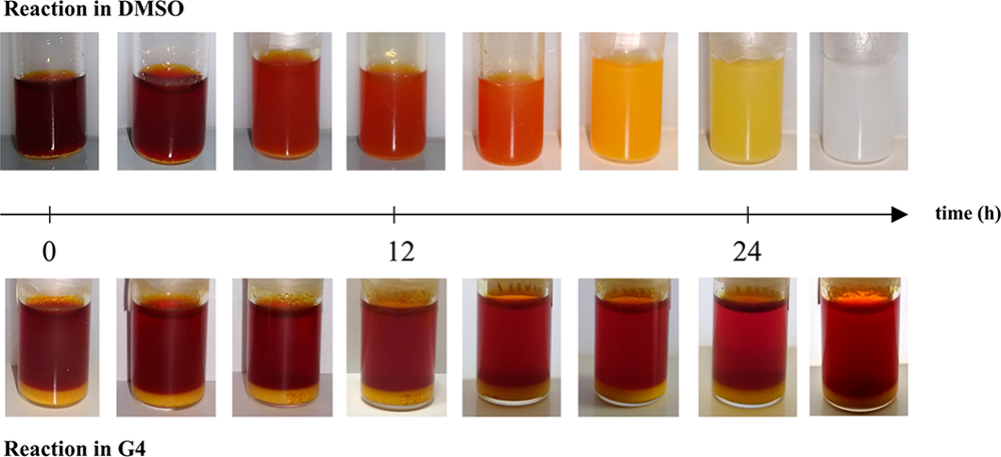
White light digital images of 1 mL of solution I_2_ 25 mM + LiI 100 mM reacting with 400 μmol of commercial Li_2_O_2_ in DMSO (top) and G4 (bottom), registered during 28 h of reaction.

To shed light on the interplay between solvation properties and the use of LiI as a redox mediator, we analyzed the chemistry of Li_2_O_2_ with I_3_^–^ in G4 and DMSO. Our aim is to check the ability of the oxidated form of the RM to promote the OER and the possible release of ^1^O_2_. The white light image evolution of Li_2_O_2_/I_2_/LiI/solvent solutions at room temperature is shown in Figure 4.

**Figure 5:**
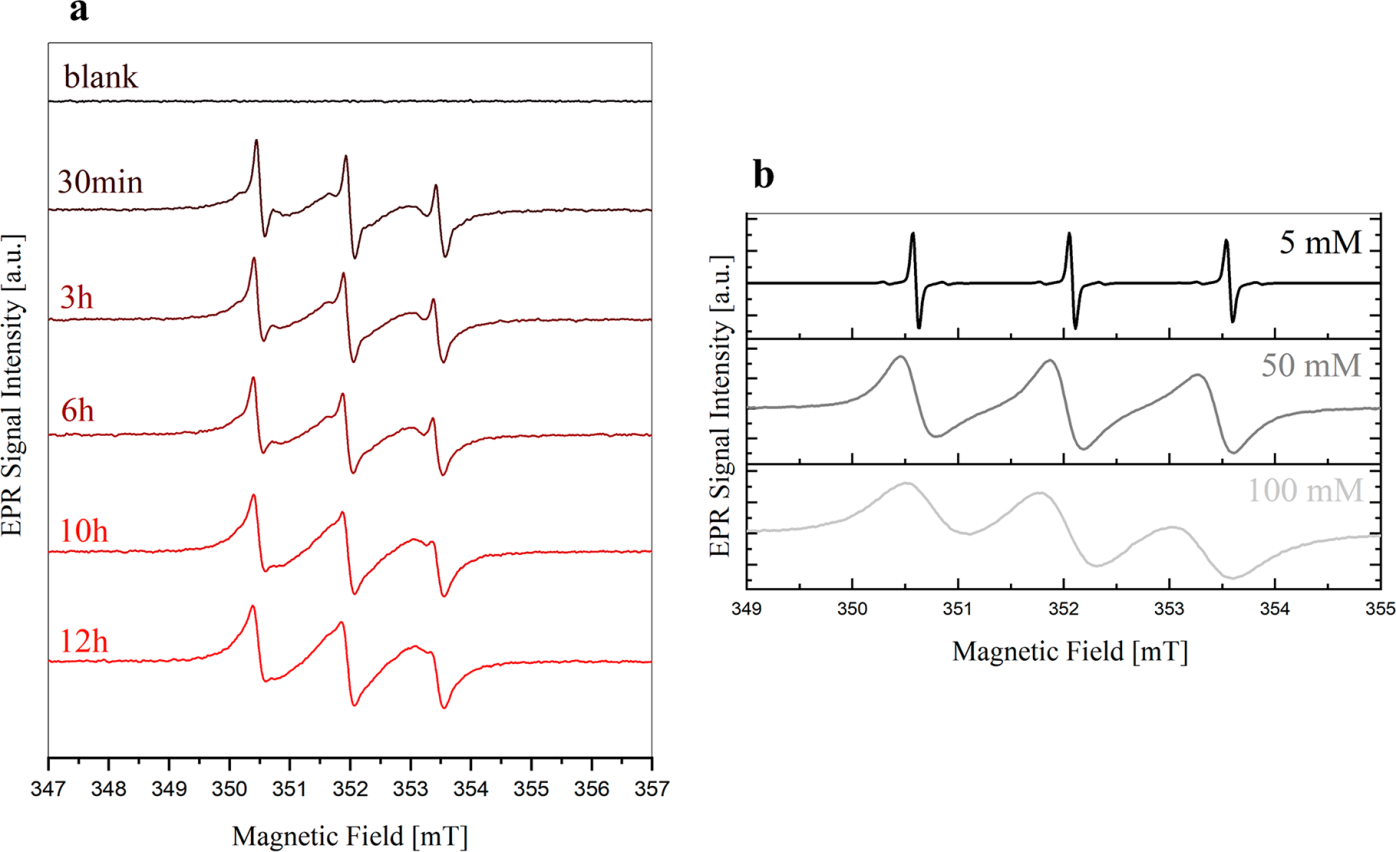
(a) EPR spectra of a solution of I_2_ 50 mM + LiI 200 mM + 80 mM 4-oxo-TEMP in DMSO reacting with excess Li_2_O_2_ for 12 h. (b) EPR spectra of solutions of 5 mM (black), 50 mM (dark gray), and 100 mM (light gray) of 4-oxo-TEMPO in DMSO.

I_2_ and LiI can be dissolved in both G4 and DMSO giving red-brownish and clear solutions, respectively, whereas lithium peroxide does not dissolve either in DMSO or in G4, thus leading to white opalescent suspensions in both solvents. As reported by Leverick et al.,^26^ mixing LiI and I_2_ with a 4:1 molar ratio leads to the complete association of I_2_ to I^–^ giving I_3_^–^. The color change of the DMSO solution (from red to white) is due to the reduction of I_3_^–^ to I^–^. On the contrary, no significant changes occur in the G4 solution. These results are consistent with the available literature: Leverick et al.^26^ proved that the kinetics of Li_2_O_2_ oxidation by I_3_^–^ is significantly faster in DMSO than in G4. A similar experiment was carried out in situ in the EPR spectrometer. During the reaction, we used the spin trap 4-oxo-TEMP, which forms the stable radical 4-oxo-TEMPO reacting selectively with ^1^O_2_, to monitor singlet oxygen evolution.^44^ No other reaction products involving the spin trap are expected.^11^ While the reduced form 4-oxo-TEMP does not have an EPR signal, the oxidized form 4-oxo-TEMPO has a unique EPR fingerprint that allows one to detect ^1^O_2_: experimental results are shown in Figure 5.

The temporal evolution of the EPR spectra of the DMSO reaction solution starting from 30 min after sample preparation until 12 h later is reported in Figure 5a. In the DMSO solution, the typical three-line signal of 4-oxo-TEMPO with a hyperfine constant of 41.5 MHz is observed from the early stages of the reaction, clearly highlighting the release of singlet oxygen that led to the oxidation of a part of the 4-oxo-TEMP to 4-oxo-TEMPO.^45^ Two partially overlapping components of the 4-oxo-TEMPO signal can be recognized, characterized by two different line widths: a well-resolved component with a line width of 0.13 mT and a broader component with a line width of 0.77 mT. The first component is the typical EPR signal attributed to 4-oxo-TEMPO in low concentration solutions, while the wider component can be attributed to regions characterized by a higher concentration of 4-oxo-TEMPO molecules, in which, due to the decreasing distance between paramagnetic centers, dipole–dipole interactions and mainly spin-exchange effects result in a broadening of the EPR line.^45^ In Figure 5b, the spectra of three solutions at known concentrations of 4-oxo-TEMPO are reported to highlight the changes in the profile shape and line width due to increasing concentrations.

The presence of both components in the DMSO solution is due to the presence of two different local 4-oxo-TEMPO environments:

(i) The broad component represents the interface between solid Li_2_O_2_ and the solution, where the radical molecules are locally more concentrated.
(ii) The narrow signal is related to noninteracting 4-oxo-TEMPO molecules diffused in solution.

The signal of the sample in DMSO initially shows mainly the narrow component, with a small contribution from the broader component appearing as a peak shoulder. Subsequently, the EPR signal intensity of the narrower component remains stable, whereas the signal intensity of the broad component increases with time. This behavior is explained by the increase of the amount of 4-oxo-TEMPO following the Li_2_O_2_ oxidation, that implies the increase of its local concentration in proximity to the Li_2_O_2_ surface. The narrower-width signal, on the other hand, can be attributed to diluted 4-oxo-TEMPO molecules that diffused in the solution during the early stages of reaction. Owing to this, this component does not change in intensity with the ongoing reaction.

**Figure 6:**
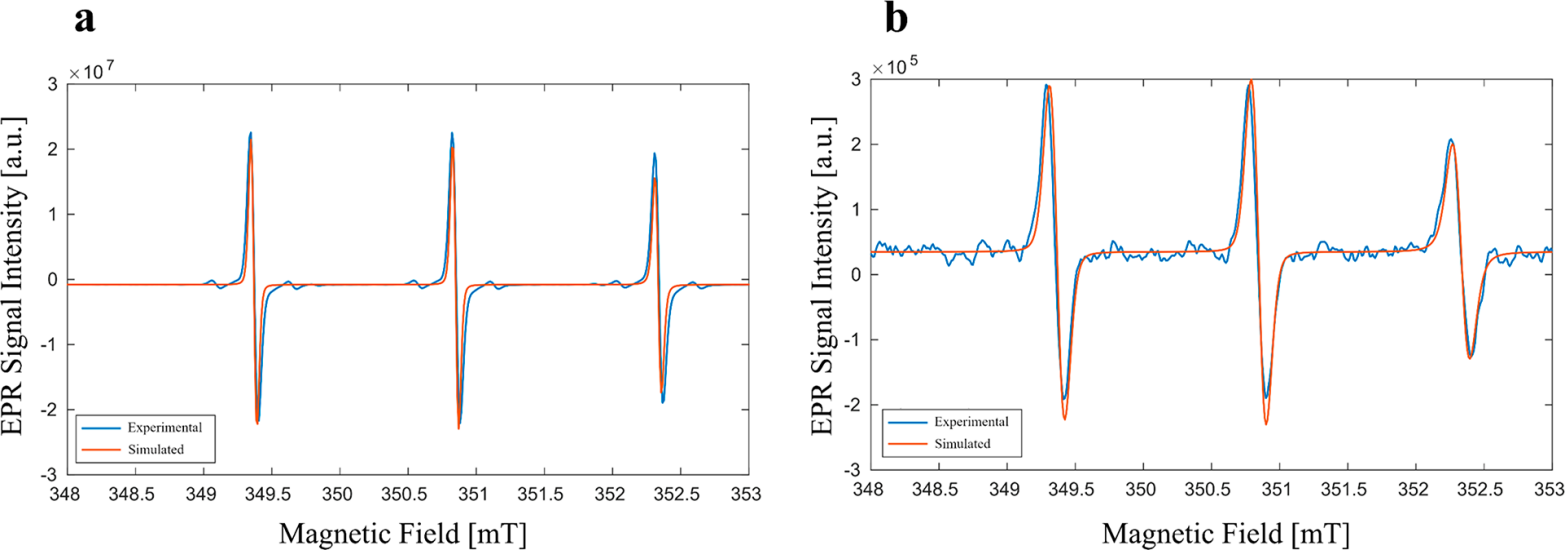
(a) Experimental (blue) and simulated (red) spectra of a 5 mM solution of 4-oxo-TEMPO in DMSO. (b) Experimental (blue) and simulated (red) spectra of the reaction solution in DMSO measured after the complete reaction.

To estimate the percentage of singlet oxygen produced during the reaction, another EPR test in DMSO was performed at the end of the reaction, 72 h after preparation. In this case, only the component with smaller line width appears (lw = 0.13 mT), since the sample was accurately mixed shortly before the EPR measurement. In Figure 6b, the acquired and simulated spectra of the sample are shown. For concentration determination, the sample was compared with the EPR spectrum of 4-oxo-TEMPO at the known concentration of 5 mM in DMSO, and the acquired and simulated spectra for this reference solution are reported in Figure 6a.

A concentration of 1 mM 4-oxo-TEMPO in the reaction solution is estimated from the ratio of the double integrals of the two simulated spectra. Assuming that 1 mM 4-oxo-TEMPO is due to the formation of 1 mM ^1^O_2_, it constitutes that at least 2% of the total molecular oxygen evolved.

**Figure 7:**
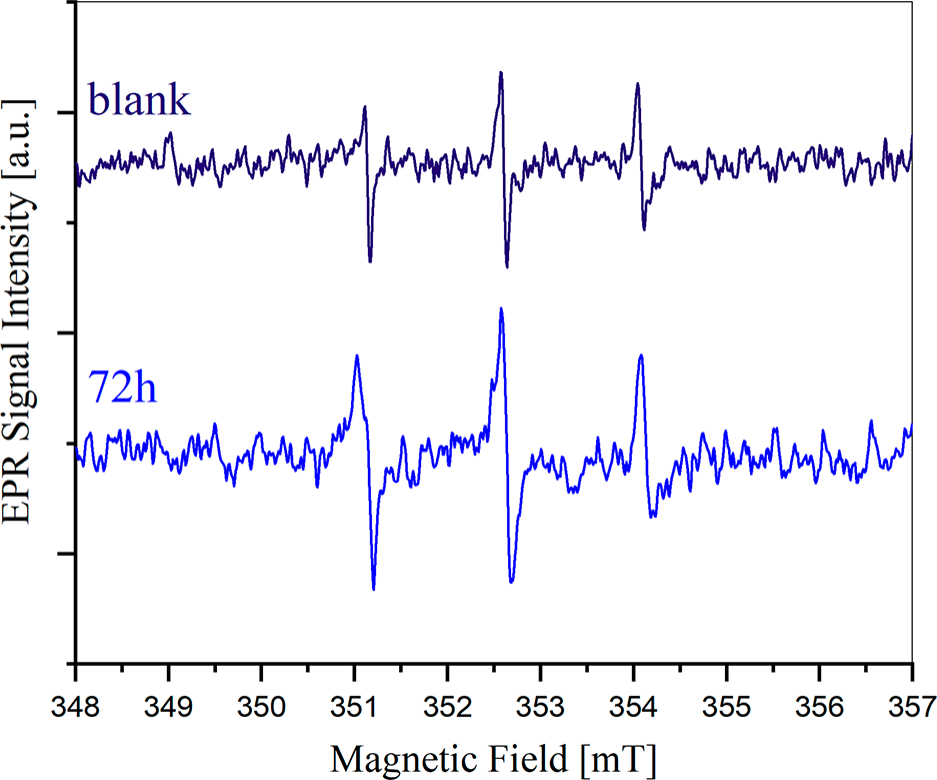
EPR spectrum of a reference solution of 80 mM 4-oxo-TEMP in G4 (dark blue) and EPR spectrum of a solution of I_2_ 50 mM + LiI 200 mM + 80 mM 4-oxo-TEMP with excess Li_2_O_2_ after 72 h of reaction (light blue).

Furthermore, it must be considered that not all of the singlet oxygen formed in the chemical reaction contributes to the formation of TEMPO molecules, as different singlet oxygen deactivation processes occur in the solution.^11^ Therefore, this estimation provides only the lower limit of ^1^O_2_ released. This value is largely beyond any thermodynamic prediction based on the ^3^O_2_ → ^1^O_2_ Gibbs energy of formation (0.97 eV) and suggests a direct reaction channel to form ^1^O_2_ promoted by the RM. Turning to the G4-based electrolyte, the EPR spectrum of the lithium peroxide/iodine solution after 72 h is shown in Figure 7.

In this case, the intensity of the signal is comparable to the blank, indicating that no significant singlet oxygen evolution occurred. In fact, the benchmark solution of 4-oxo-TEMP 80 mM in G4 reported as reference shows at very low intensities the typical 4-oxo-TEMPO signal caused by small amounts of impurities in the commercial 4-oxo-TEMP.^11^ Overall, our results demonstrate that singlet oxygen evolution is strongly dependent on the nature of the aprotic solvent.

## Discussion

In the previous section, we reported direct evidence of the alteration of the aLOBs reversibility and degradation reactivity, at both the positive and negative electrodes, depending on the electrolyte formulation. This finding has a direct match in the change of the spontaneous chemical reactivity of the lithium peroxide with iodine in DMSO and G4, as proven by white light imaging and EPR spectroscopy. The stoichiometries of the chemical OERs promoted by lithium triiodide are given by
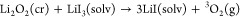
R1
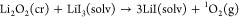
R2where Li_2_O_2_(cr), LiI_3_(solv), LiI(solv), ^3^O_2_(g), and ^1^O_2_(g) are solid lithium peroxide, solvated lithium triiodide, solvated lithium iodide, and molecular oxygen in his triplet or singlet state, respectively. Given the energy difference between triplet and singlet state molecular oxygen (i.e., 0.97 eV),^46^reaction R2 has a much more unfavorable thermodynamics compared to reaction R1.

**Figure 8:**
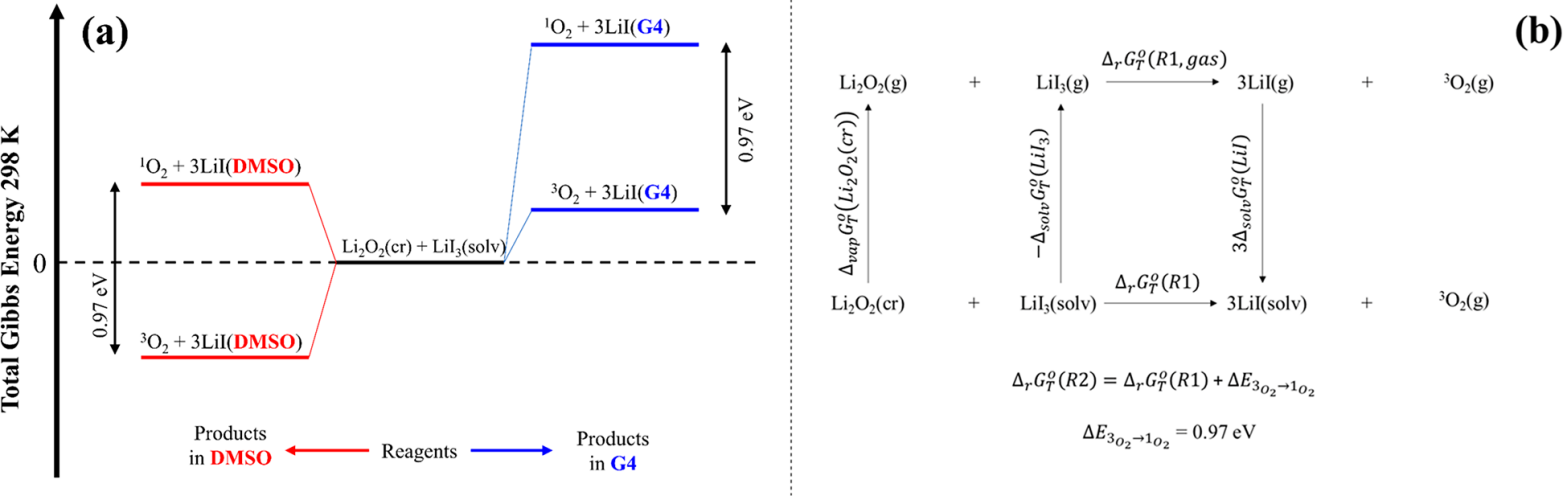
(a) Qualitative landscape of the potential energy surfaces of the heterogeneous reaction Li_2_O_2_(cr) + LiI_3_(solv) → 3LiI(solv) + O_2_(g) in DMSO (red) and G4 (blue); (b) thermochemical equations of reactions R1 and R2.

Experimentally, reactions R1 and R2 activate thanks to a strong driving force in DMSO, whereas in G4 our results suggest an unfavorable energetic landscape. Both thermodynamic reaction paths in both electrolytes are sketched in Figure 8a as qualitative potential energy surfaces of the heterogeneous reaction Li_2_O_2_(cr) + LiI_3_(solv) → 3LiI(solv) + O_2_(g).

As discussed above, in G4, either reaction R1 or R2 shows unfavorable thermodynamics: this evidence must reflect an increase of total Gibbs energy passing from reagents to products. On the contrary in DMSO, both reactions occur, suggesting a favorable driving force: in this case, the total Gibbs energy must show a decreasing trend from reagents to products. Therefore, the key factor affecting the different energetic landscapes is the solvation thermodynamics of lithium iodide and lithium triiodide. The thermochemical equations that describe reactions R1 and R2 and their dependence by solvation thermodynamics are represented in Figure 8b. The Gibbs energy of the heterogeneous reaction R1, i.e., Δ_*r*_*G*_*T*_^o^(R1), is given by
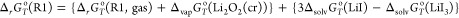
1where all standard Gibbs energy of reaction, vaporization, or solvation symbols correspond to those shown in Figure 8b.

Overall Δ_*r*_*G*_*T*_^o^(R1) depends on the two sums grouped in braces: its sign, either positive or negative, is given by the balance of these two addends. The first sum in eq 1 is a positive quantity, i.e., {Δ_*r*_*G*_*T*_^o^(R1,gas) + Δ_vap_*G*_*T*_^o^(Li_2_O_2_(cr))} > 0, being Δ_*r*_*G*_*T*_^o^(R1,gas) driven by the unfavorable standard reaction entropy as well as (Δ_vap_*G*_*T*_^o^(Li_2_O_2_(cr)). Furthermore, it is independent of the solvation media. The second quantity in eq 1, i.e., {3Δ_solv_*G*_*T*_^o^(LiI) – Δ_solv_*G*_*T*_^o^(LiI_3_)}, is the weighted sum of the Gibbs energies of solvation of lithium iodide and lithium triiodide. Both LiI and LiI_3_ being ionic molecules, both Δ_solv_*G*_*T*_^o^(LiI) and Δ_solv_*G*_*T*_^o^(LiI_3_) are negative quantities in polar solvents, even with small dielectric constants like G4. Thus, the sign of the overall Gibbs energy of the heterogeneous reaction R1 is driven by the balance between these two terms:
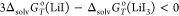
2
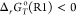

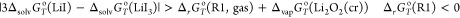
3
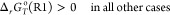
4

Generally speaking, Δ_*r*_*G*_*T*_^o^(R1) < 0 is true only if 3Δ_solv_*G*_*T*_^o^(LiI_3_) < Δ_solv_*G*_T_^o^(LiI_3_). As a consequence, the activation of R1 in DMSO implies a much more negative value for the difference 3Δ_solv_*G*_*T*_^o^(LiI) – 3Δ_solv_*G*_*T*_^o^(LiI_3_) compared to G4: this thermodynamic constraint implies a much better solvation of LiI with respect to LiI_3_. Unavoidably, the decrease of Δ_*r*_*G*_*T*_^o^(R1) to negative values also downshifts the thermodynamics of reaction R2, i.e., Δ_*r*_*G*_*T*_^o^(R2), thus enhancing the detrimental release of the singlet oxygen.

## Conclusion

In this work, we demonstrated experimentally that the OER mediated by iodine is chemically hindered in a G4-based electrolyte whereas in DMSO a thermodynamic push is provided by the more favorable solvation thermodynamics. Unfortunately, the onset of reaction R1 in DMSO follows in parallel a remarkable release of singlet molecular oxygen. In aLOBs, iodine promotes the OER in both electrolytes, likely helped by overpotentials in G4. However, in DMSO-based electrolytes, the massive release of singlet oxygen leads to rapid cell failure.

Our findings suggest that the redox mediation by iodine in aLOBs can be tuned by altering the dielectric constant of the electrolyte solvent, thus providing more effective OER kinetics. On the other hand, this thermodynamic push must be carefully balanced to minimize the concurrent reactive channel that leads to the release of singlet molecular oxygen. In fact, in DMSO, the activation of a spontaneous chemical OER promoted by LiI_3_ quickly leads to accumulation of degradation byproducts resulting from the spontaneous reactivity of singlet oxygen with all the constituents of the cell.

It is important to stress that our experimental evidence highlights the subtle unexpected origin of the degradation reactivity occurring in DMSO. On one hand, the change in the solvent polarity has a direct impact on the OER thermodynamics promoted by the same RM compared to G4. On the other hand, an undesired consequence is the parallel formation of ^1^O_2_. This detrimental process activates multiple parasitic reactivities, leading to cell death via the accumulation of byproducts in any compartment of the cell.

Overall, our analysis suggests that the choice of the redox mediator and the electrolyte must be balanced together in aLOBs in order to find a favorable equilibrium between OER activation and the release of singlet molecular oxygen.

## Supporting Information Available

Stoichiometric calculations; potential profiles; Raman spectra (PDF)

## References

[ref1] Kwak W. J.; Rosy N.; Sharon D.; Xia C.; Kim H.; Johnson L. R.; Bruce P. G.; Nazar L. F.; Sun Y. K.; Frimer A. A.; Noked M.; Freunberger S. A. Lithium-Oxygen Batteries and Related Systems: Potential, Status, and Future. Chem. Rev. 2020, 120, 6626–6683. 10.1021/acs.chemrev.9b00609. 32134255

[ref2] Wang Y.; Lu Y. Nonaqueous Lithium-Oxygen batteries: Reaction mechanism and critical open questions. Energy Storage Mater. 2020, 28, 235–246. 10.1016/j.ensm.2020.03.007.

[ref3] Varzi A.; Thanner K.; Scipioni R.; Di Lecce D.; Hassoun J.; Dörfler S.; Altheus H.; Kaskel S.; Prehal C.; Freunberger S. A. Current status and future perspectives of lithium metal batteries. J. Power Sources 2020, 480, 228803 10.1016/j.jpowsour.2020.228803.

[ref4] Bi X.; Amine K.; Lu J. The importance of anode protection towards lithium oxygen batteries. J. Mater. Chem. A 2020, 8, 3563–3573. 10.1039/C9TA12414D.

[ref5] Mahne N.; Fontaine O.; Thotiyl M. O.; Wilkening M.; Freunberger S. A. Mechanism and performance of lithium-oxygen batteries - a perspective. Chem. Sci. 2017, 8, 6716–6729. 10.1039/C7SC02519J. 29147497 PMC5643885

[ref6] Hong Y. S.; Zhao C. Z.; Xiao Y.; Xu R.; Xu J. J.; Huang J. Q.; Zhang Q.; Yu X.; Li H. Safe Lithium-Metal Anodes for Li-O2 Batteries: From Fundamental Chemistry to Advanced Characterization and Effective Protection. Batter. Supercaps 2019, 2, 638–658. 10.1002/batt.201900031.

[ref7] Aurbach D.; McCloskey B. D.; Nazar L. F.; Bruce P. G. Advances in understanding mechanisms underpinning lithium-air batteries. Nat. Energy 2016, 1, 16128 10.1038/nenergy.2016.128.

[ref8] Mahne N.; Schafzahl B.; Leypold C.; Leypold M.; Grumm S.; Leitgeb A.; Strohmeier G. A.; Wilkening M.; Fontaine O.; Kramer D.; Slugovc C.; Borisov S. M.; Freunberger S. A. Singlet oxygen generation as a major cause for parasitic reactions during cycling of aprotic lithium-oxygen batteries. Nat. Energy 2017, 2, 17036 10.1038/nenergy.2017.36.

[ref9] Mourad E.; Petit Y. K.; Spezia R.; Samojlov A.; Summa F. F.; Prehal C.; Leypold C.; Mahne N.; Slugovc C.; Fontaine O.; Brutti S.; Freunberger S. A. Singlet oxygen from cation driven superoxide disproportionation and consequences for aprotic metal-O_2_ batteries. Energy Environ. Sci. 2019, 12, 2559–2568. 10.1039/C9EE01453E.

[ref10] Lee H. W.; Kim J. Y.; Kim J. E.; Jo Y. J.; Dewar D.; Yang S.; Gao X.; Bruce P. G.; Kwak W. J. Effect of singlet oxygen on redox mediators in lithium-oxygen batteries. J. Mater. Chem. A 2023, 11, 16003–16008. 10.1039/D3TA01284K.

[ref11] Wandt J.; Jakes P.; Granwehr J.; Gasteiger H. A.; Eichel R. A. Singlet Oxygen Formation during the Charging Process of an Aprotic Lithium-Oxygen Battery. Angew. Chemie Int. Ed. 2016, 55, 6892–6895. 10.1002/anie.201602142. 27145532

[ref12] Carboni M.; Brutti S.; Marrani A. G. Surface Reactivity of a Carbonaceous Cathode in a Lithium Triflate/Ether Electrolyte-Based Li-O_2_ Cell. ACS Appl. Mater. Interfaces 2015, 7, 21751–21762. 10.1021/acsami.5b05202. 26375042

[ref13] Bergner B. J.; Schürmann A.; Peppler K.; Garsuch A.; Janek J. TEMPO: A mobile catalyst for rechargeable Li-O_2_ batteries. J. Am. Chem. Soc. 2014, 136, 15054–15064. 10.1021/ja508400m. 25255228

[ref14] Petit Y. K.; Leypold C.; Mahne N.; Mourad E.; Schafzahl L.; Slugovc C.; Borisov S. M.; Freunberger S. A. DABCOnium: An Efficient and High-Voltage Stable Singlet Oxygen Quencher for Metal-O_2_ Cells. Angew. Chemie Int. Ed. 2019, 58, 6535–6539. 10.1002/anie.201901869. PMC6563493 30884063

[ref15] Kwak W. J.; Hirshberg D.; Sharon D.; Frimer A. A.; Jung H. G.; Aurbach D.; Sun Y. K. Li-O_2_ cells with LiBr as an electrolyte and a redox mediator. Energy Environ. Sci. 2016, 9, 2334–2345. 10.1039/C6EE00700G.

[ref16] Kwak W. J.; Hirshberg D.; Sharon D.; Shin H. J.; Afri M.; Park J. B.; Garsuch A.; Chesneau F. F.; Frimer A. A.; Aurbach D.; Sun Y. K. Understanding the behavior of Li-oxygen cells containing LiI. J. Mater. Chem. A 2015, 3, 8855–8864. 10.1039/C5TA01399B.

[ref17] Petit Y. K.; Mourad E.; Prehal C.; Leypold C.; Windischbacher A.; Mijailovic D.; Slugovc C.; Borisov S. M.; Zojer E.; Brutti S.; Fontaine O.; Freunberger S. A. Mechanism of mediated alkali peroxide oxidation and triplet versus singlet oxygen formation. Nat. Chem. 2021, 13, 465–471. 10.1038/s41557-021-00643-z. 33723377

[ref18] Zhong H.; Wang J.; Wang Y.; He P.; Zhou H. Progress and Prospects in Redox Mediators for Highly Reversible Lithium-Oxygen Batteries: A Minireview. Energy Fuels 2021, 35, 19302–19319. 10.1021/acs.energyfuels.1c02844.

[ref19] Bi X.; Li J.; Dahbi M.; Alami J.; Amine K.; Lu J. Understanding the Role of Lithium Iodide in Lithium-Oxygen Batteries. Adv. Mater. 2022, 34, 2106148 10.1002/adma.202106148. 34854504

[ref20] Qiao Y.; Wu S.; Sun Y.; Guo S.; Yi J.; He P.; Zhou H. Unraveling the Complex Role of Iodide Additives in Li-O_2_ Batteries. ACS Energy Lett. 2017, 2, 1869–1878. 10.1021/acsenergylett.7b00462.

[ref21] Liu T.; Liu Z.; Kim G.; Frith J. T.; Garcia-Araez N.; Grey C. P. Understanding LiOH Chemistry in a Ruthenium-Catalyzed Li-O_2_ Battery. Angew. Chemie Int. Ed. 2017, 129, 16273–16278. 10.1002/ange.201709886. PMC6033020 29058366

[ref22] Burke C. M.; Black R.; Kochetkov I. R.; Giordani V.; Addison D.; Nazar L. F.; McCloskey B. D. Implications of 4 e- Oxygen Reduction via Iodide Redox Mediation in Li-O_2_ Batteries. ACS Energy Lett. 2016, 1, 747–756. 10.1021/acsenergylett.6b00328.

[ref23] Zhang X.; Dong P.; Song M. K. Advances in Lithium-Oxygen Batteries Based on Lithium Hydroxide Formation and Decomposition. Front. Chem. 2022, 10, 923936 10.3389/fchem.2022.923936. 35844634 PMC9283641

[ref24] Li Y.; Dong S.; Chen B.; Lu C.; Liu K.; Zhang Z.; Du H.; Wang X.; Chen X.; Zhou X.; Cui G. Li-O_2_ Cell with LiI(3-hydroxypropionitrile)2 as a Redox Mediator: Insight into the Working Mechanism of I- during Charge in Anhydrous Systems. J. Phys. Chem. Lett. 2017, 8, 4218–4225. 10.1021/acs.jpclett.7b01497. 28825835

[ref25] Nakanishi A.; Thomas M. L.; Kwon H. M.; Kobayashi Y.; Tatara R.; Ueno K.; Dokko K.; Watanabe M. Electrolyte Composition in Li/O_2_ Batteries with LiI Redox Mediators: Solvation Effects on Redox Potentials and Implications for Redox Shuttling. J. Phys. Chem. C 2018, 122, 1522–1534. 10.1021/acs.jpcc.7b11859.

[ref26] Leverick G.; Tułodziecki M.; Tatara R.; Bardé F.; Shao-Horn Y. Solvent-Dependent Oxidizing Power of LiI Redox Couples for Li-O2 Batteries. Joule 2019, 3, 1106–1126. 10.1016/j.joule.2018.12.014.

[ref27] Wang A.; Wu X.; Zou Z.; Qiao Y.; Wang D.; Xing L.; Chen Y.; Lin Y.; Avdeev M.; Shi S. The Origin of Solvent Deprotonation in LiI-added Aprotic Electrolytes for Li-O2 Batteries. Angew. Chemie - Int. Ed. 2023, 62, 62 10.1002/anie.202217354. 36749300

[ref28] He W.; Kim H. K.; Wamer W. G.; Melka D.; Callahan J. H.; Yin J. J. Photogenerated charge carriers and reactive oxygen species in ZnO/Au hybrid nanostructures with enhanced photocatalytic and antibacterial activity. J. Am. Chem. Soc. 2014, 136, 750–757. 10.1021/ja410800y. 24354568

[ref29] He W.; Jia H.; Cai J.; Han X.; Zheng Z.; Wamer W. G.; Yin J. J. Production of Reactive Oxygen Species and Electrons from Photoexcited ZnO and ZnS Nanoparticles: A Comparative Study for Unraveling their Distinct Photocatalytic Activities. J. Phys. Chem. C 2016, 120, 3187–3195. 10.1021/acs.jpcc.5b11456.

[ref30] Chai J. D.; Head-Gordon M. Long-range corrected hybrid density functionals with damped atom-atom dispersion corrections. Phys. Chem. Chem. Phys. 2008, 10, 6615–6620. 10.1039/b810189b. 18989472

[ref31] Neese F. Software update: The ORCA program system—Version 5.0. Wiley Interdiscip. Rev. Comput. Mol. Sci. 2022, 12, e1606 10.1002/wcms.1606.

[ref32] Stoll S.; Schweiger A. EasySpin, a comprehensive software package for spectral simulation and analysis in EPR. J. Magn. Reson. 2006, 178, 42–55. 10.1016/j.jmr.2005.08.013. 16188474

[ref33] Chen Y.; Gao X.; Johnson L. R.; Bruce P. G. Kinetics of lithium peroxide oxidation by redox mediators and consequences for the lithium-oxygen cell. Nat. Commun. 2018, 9 (1), 767 10.1038/s41467-018-03204-0. 29472558 PMC5823882

[ref34] Bergner B. J.; Hofmann C.; Schürmann A.; Schröder D.; Peppler K.; Schreiner P. R.; Janek J. Understanding the fundamentals of redox mediators in Li-O_2_ batteries: a case study on nitroxides. Phys. Chem. Chem. Phys. 2015, 17, 31769–31779. 10.1039/C5CP04505C. 26563563

[ref35] Ahmadiparidari A.; Fuladi S.; Majidi L.; Plunkett S.; Sarnello E.; Gholivand H.; Hemmat Z.; Rastegar S.; Misal S. N.; Jimenez N.; Redfern P. C.; Wen J. Enhancing the performance of lithium oxygen batteries through combining redox mediating salts with a lithium protecting salt. J. Power Sources 2021, 491, 229506 10.1016/j.jpowsour.2021.229506.

[ref36] Li D.; Kang Z.; Sun H.; Wang Y.; Xie H.; Liu J.; Zhu J. A bifunctional MnxCo_3_-xO_4_-decorated separator for efficient Li-LiI-O_2_ batteries: A novel strategy to promote redox coupling and inhibit redox shuttling. Chem. Eng. J. 2022, 428, 131105 10.1016/j.cej.2021.131105.

[ref37] Skripkin M. Y.; Lindqvist-Reis P.; Abbasi A.; Mink J.; Persson I.; Sandström M. Vibrational spectroscopic force field studies of dimethyl sulfoxide and hexakis(dimethyl sulfoxide)scandium(III) iodide, and crystal and solution structure of the hexakis(dimethyl sulfoxide)scandium(III) ion. Dalt. Trans. 2004, 23, 4038–4049. 10.1039/B413486A. 15558131

[ref38] Parker S. F.; Revill-Hivet E. J.; Nye D. W.; Gutmann M. J. Structure and vibrational spectroscopy of lithium and potassium methanesulfonates. R. Soc. Open Sci. 2020, 7, 200776 10.1098/rsos.200776. 32874662 PMC7428240

[ref39] Sharon D.; Afri M.; Noked M.; Garsuch A.; Frimer A. A.; Aurbach D. Oxidation of dimethyl sulfoxide solutions by electrochemical reduction of oxygen. J. Phys. Chem. Lett. 2013, 4, 3115–3119. 10.1021/jz4017188. 26291224

[ref40] Papke B. L.; Ratner M. A.; Shriver D. F. Vibrational spectroscopy and structure of polymer electrolytes, poly(ethylene oxide) complexes of alkali metal salts. J. Phys. Chem. Solids 1981, 42, 493–500. 10.1016/0022-3697(81)90030-5.

[ref41] Kim K.; Kuhn L.; Alabugin I. V.; Hallinan D. T. Lithium Salt Dissociation in Diblock Copolymer Electrolyte Using Fourier Transform Infrared Spectroscopy. Front. Energy Res. 2020, 8, 569442 10.3389/fenrg.2020.569442. 32073855

[ref42] Kam W.; Liew C. W.; Lim J. Y.; Ramesh S. Electrical, structural, and thermal studies of antimony trioxide-doped poly(acrylic acid)-based composite polymer electrolytes. Ionics 2014, 20, 665–674. 10.1007/s11581-013-1012-0.

[ref43] Haas R.; Janek J. The Influence of Oxygen Dissolved in the Liquid Electrolyte on Lithium Metal Anodes. J. Electrochem. Soc. 2022, 169, 110527 10.1149/1945-7111/ac9d6b.

[ref44] Nardi G.; Manet I.; Monti S.; Miranda M. A.; Lhiaubet-Vallet V. Scope and limitations of the TEMPO/EPR method for singlet oxygen detection: The misleading role of electron transfer. Free Radic. Biol. Med. 2014, 77, 64–70. 10.1016/j.freeradbiomed.2014.08.020. 25236741

[ref45] Wessig M.; Spitzbarth M.; Drescher M.; Winter R.; Polarz S. Multiple scale investigation of molecular diffusion inside functionalized porous hosts using a combination of magnetic resonance methods. Phys. Chem. Chem. Phys. 2015, 17, 15976–15988. 10.1039/C5CP01369K. 26027653

[ref46] Pierini A.; Brutti S.; Bodo E. Superoxide Anion Disproportionation Induced by Li^+^ and H^+^: Pathways to ^1^O_2_ Release in Li-O_2_ Batteries. ChemPhysChem 2020, 21, 2060–2067. 10.1002/cphc.202000318. 32667121

