# Supplementary material for: Insights into the LiI Redox Mediation in Aprotic Li–O2 Batteries: Solvation Effects and Singlet Oxygen Evolution
Source: ACS Appl Mater Interfaces. 2023 Dec 13;15(51):59348–57. doi: 10.1021/acsami.3c12330 (PMC10755701; doi:10.1021/acsami.3c12330)
Supplement: Supplementary file 1 — am3c12330_si_001.pdf [file am3c12330_si_001.pdf]

Supporting Information

Insights into the LiI Redox Mediation in Aprotic Li-O_2 Batteries: Solvation Effects and Singlet Oxygen Evolution

*Angelica Petrongari[a], Vanessa Piacentini[a], Adriano Pierini[a], Paola Fattibene[b], Cinzia De Angelis[b], Enrico Bodo[a], Sergio Brutti[a,c,d]**

[a] Department of Chemistry, Sapienza University of Rome, P.le Aldo Moro 5, Rome, 00185, Italy

[b] Core Facilities, Istituto Superiore di Sanità, Viale Regina Elena 299, Rome, 00161, Italy

[c] CNR-ISC, Consiglio Nazionale Delle Ricerche, Istituto Dei Sistemi Complessi, Rome, 00185, Italy

[d] GISEL - Centro di Riferimento Nazionale per i Sistemi di Accumulo Elettrochimico di Energia, Florence, 50121, Italy

*Email: sergio.brutti@uniroma1.it

In the Figure S1 we outline our approach to decouple the fraction of charge exchanged upon reduction that is due to the reduction of excess triiodide or by the ORR.

In the Figure S1, Q_i is the capacity originated by the reduction of I_3^- at cycle i, whereas $\Delta Q_i = Q_{lim} - Q_i$ is the capacity directly related to the ORR.

The total cumulative capacity due to the ORR is therefore:

$$\Delta Q_{tot} = \sum_{i=1}^{200} \Delta Q_i = n_{O_2}^{cum} \cdot 2 \cdot F = 7.97\, mAh\, cm^{-2}$$

Being the cathode surface S = 1,77 cm^2, the number of moles of molecular oxygen necessary to deliver this cumulative discharge capacity is:

$$n_{O_2}^{cum} = \frac{Q_{tot}\left[\frac{mAh}{cm^2}\right] \cdot S[cm^2] \cdot 3.6\,[\frac{C}{mAh}]}{2F[\frac{C}{mol}]} = 0.26\, mmol$$

Being the partial pressure of O_2 in the cell equal to 1 bar, the cell head volume 0.0043 L, at room temperature, the number of moles of molecular oxygen filled in the cell before cycling is:

$$n_{O_2}^{max} = \frac{P\,[bar] \cdot V[L]}{R\,[L\, bar\, K^{-1}\, mol^{-1}] \cdot T(K)} = 0.18\, mmol$$

As a matter of fact, $n_{O_2}^{cum} > n_{O_2}^{max}$: this unavoidably implies the occurrence of the OER to regenerate the $^3O_2$ reduced upon discharge.

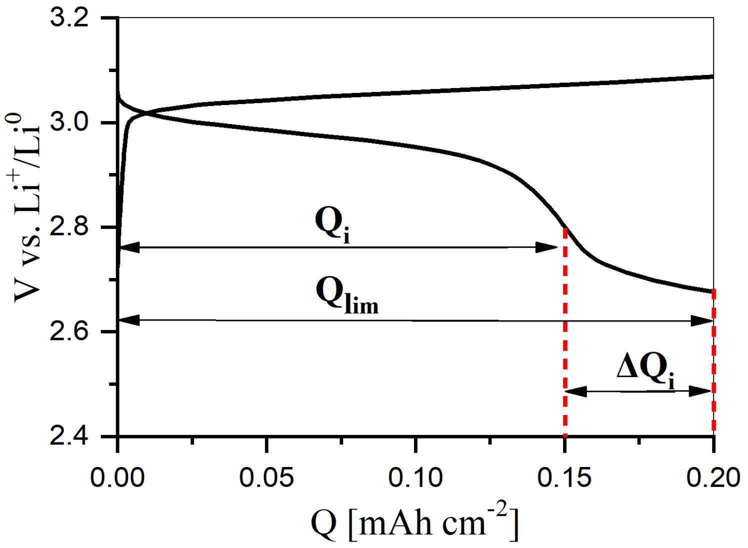

Figure S1: *schematic representation of the decoupling of discharge capacity owed to the reduction of excess triiodide and to ORR.*

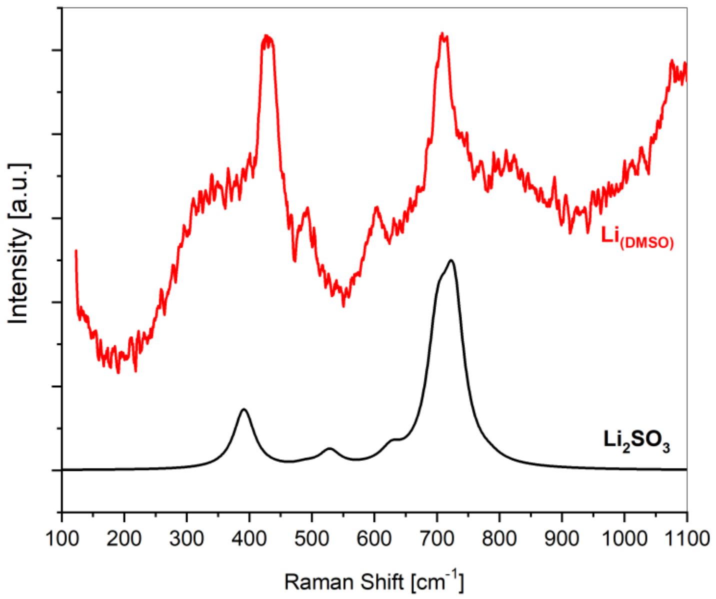

Figure S2: *Experimental Raman spectrum of $Li_{(DMSO)}$ and simulated Raman spectrum of Li_2SO_3.*
